# Accumulation of ^123^I-Ioflupane Is a Useful Marker of the Efficacy of Selegiline Monotherapy in Drug-Naïve Parkinson’s Disease

**DOI:** 10.3389/fnagi.2017.00321

**Published:** 2017-09-29

**Authors:** Hidetomo Murakami, Tetsuhito Nohara, Masanobu Uchiyama, Yoshiyuki Owan, Akinori Futamura, Azusa Shiromaru, Setsuro Tsukada, Yu Saito, Takeshi Kuroda, Satoshi Yano, Seiichiro Ishigaki, Hirotaka Katoh, Jiro Munechika, Yoshimitsu Ohgiya, Takehiko Gokan, Kenjiro Ono

**Affiliations:** ^1^Department of Neurology, School of Medicine, Showa University, Tokyo, Japan; ^2^Department of Neurology, Showa University Northern Yokohama Hospital, Yokohama, Japan; ^3^Department of Neurology, Fujiyoshida Municipal Hospital, Fujiyoshida, Japan; ^4^Department of Radiology, School of Medicine, Showa University, Tokyo, Japan

**Keywords:** Parkinson disease, selegiline monotherapy, monoamine oxidase inhibitor, ^123^I-Ioflupane SPECT, ^123^I-MIBG myocardial scintigraphy

## Abstract

**Background**: Selegiline enhances the patient’s endogenous dopamine by inhibiting dopamine metabolism. The efficacy of selegiline monotherapy for drug-naïve Parkinson’s disease (PD) patients may depend on the degree of dopaminergic neuronal degeneration. ^123^I-Ioflupane single photon emission computed tomography (SPECT) and ^123^I-meta-iodobenzylguanidine (MIBG) myocardial scintigraphy are diagnostic methods to assess the pharmacological and pathological changes in PD.

**Objective**: We examined the utility of these imaging methods to predict the efficacy of selegiline monotherapy for motor symptoms in drug-naïve PD patients.

**Methods**: We observed the efficacy of selegiline monotherapy in 28 drug-naïve PD patients and compared the improvement in motor function and the imaging findings. These patients received selegiline monotherapy, and the amount was increased to the optimal dose in clinical practice. Motor function was assessed using the Unified Parkinson’s Rating Scale (UPDRS) at baseline and at the stable dose. Imaging was performed before treatment, and the striatal Specific Binding Ratio (SBR) of the ^123^I-Ioflupane SPECT and the Heart-to-Mediastinum (H/M) ratio of the ^123^I-MIBG myocardial scintigraphy were calculated. Both ratios were compared with improvements in scores for motor assessment using Pearson’s correlation coefficient.

**Results**: The mean UPDRS part III score significantly improved with at least 5.0 mg/day of selegiline. Further dose escalation did not improve the mean motor score. The percent improvement in the motor score from baseline showed a significant negative correlation with the SBR of average of the right and left striatum, but not with the H/M ratio. Multiple regression analysis using patient’s background factors showed that percent improvement in the UPDRS part III score directly correlate with the SBR (*p* = 0.04), but not with the age (*p* = 0.72), disease duration (*p* = 0.31), baseline UPDRS part III (*p* = 0.77) and the drug dose (*p* = 0.26).

**Conclusion**: PD patients with a lower accumulation of ^123^I-Ioflupane in the striatum can have greater improvement with selegiline monotherapy.

## Introduction

Since the 1960s, levodopa has been successfully used to treat motor symptoms in Parkinson’s disease (PD). Subsequently, other drugs, such as dopamine agonists, monoamine oxidase (MAO) inhibitors and others, were introduced into clinical practice. For the initial treatment for drug-naïve PD patients, a dopamine agonist and levodopa are frequently used. Selegiline, a MAO type B (MAO-B) inhibitor, is also recommended for the initial treatment in drug-naïve PD patients (Jankovic and Poewe, 2012). The initial treatment with selegiline delays the need for levodopa introduction (Pålhagen et al., 1998). The PD MED study published in 2014 showed very small but persistent better mobility scores were recorded when treatment was initiated with MAO-B inhibitor than with dopamine agonists (PD Med Collaborative Group et al., 2014). Based on its pharmacology, selegiline enhances the patient’s endogenous dopamine by inhibiting MAO-B without introducing exogenous dopamine. Thus, the efficacy seems to depend on the patient’s ability to produce dopamine in the degenerating nigrostriatal systems. In clinical practice, the efficacy for drug-naïve PD patients varies depending on the patient. At the moment, responders of selegiline monotherapy can’t be detected before the medication.

^123^I-Ioflupane Single Photon Emission Computed Tomography (SPECT) detects degeneration of the dopamine transporter in the striatum (Booij et al., 1997). Decreased striatal radiological uptake in such imaging indicates a loss of nigrostriatal dopaminergic neurons (Colloby et al., 2012). Thus ^123^I-Ioflupane SPECT is used to assess degeneration of nigrostriatal dopaminergic neurons (Kägi et al., 2010). On the other hand, ^123^I-meta-iodobenzylguanidine (MIBG) myocardial scintigraphy evaluates cardiac sympathetic nerve function and is useful for differentiating PD from other Parkinsonian syndromes (Orimo et al., 2012). Furthermore, it is a biomarker for the presence of Lewy bodies, the main pathological finding in PD (Orimo et al., 2016), and the Heart-to-Mediastinum (H/M) ratio for this scintigraphy reflects the severity of motor function in PD (Saiki et al., 2004). Therefore these imaging biomarkers have a possibility to correlate with patient’s endogenous dopamine level and to detect responders of selegiline monotherapy. Here, we examined whether the ^123^I-Ioflupane SPECT and ^123^I-MIBG myocardial scintigraphy predict efficacy of selegiline monotherapy for motor symptoms in drug-naïve PD patients.

## Materials and Methods

### Subjects

Twenty-eight, drug-naïve PD patients were recruited from outpatient and inpatient groups diagnosed at Showa University Hospital and Showa University East Hospital, Tokyo, Japan. The diagnosis of PD was made using the United Kingdom PD Society Brain Bank criteria (Gibb and Lees, 1988). Patients were first diagnosed by clinical history and by neurological findings before medication, and then confirmed as PD patients after demonstrating improved motor symptoms on administration of dopaminergic drugs. The motor symptoms of all, except one, of the patients improved with selegiline, and their diagnosis were unchanged. The treatment for the other patient was changed to ropinirole and then later levodopa, and her symptoms improved. Thus, she was diagnosed as PD. Data for this patient were obtained before use of all drugs except selegiline. All patients had a Mini-Mental State Examination (MMSE) score of 25 or more, but none had evidence of core clinical features of Dementia with Lewy Bodies (DLB), such as fluctuating cognition with pronounced variations in attention and alertness, recurrent visual hallucinations that are typically well formed and detailed, and REM sleep behavior disorder (McKeith et al., 2017). Therefore, we diagnosed PD or PD with mild cognitive impairment (MCI) in all subjects. No patient was taking an anti-dementia drug, such as either an acetylcholinesterase inhibitor or N-methyl-D-aspartic acid (NMDA) receptor antagonist. No patient was administered an anti-cholinergic drug, such as trihexyphenidyl. None had a history of an impulse control disorder. None had a disease except PD that affected motor and cognitive functions. The Ethics Committee of Showa University School of Medicine approved this study, and it was performed according to the Declaration of Helsinki. Written informed consent was obtained from all participants.

### Clinical Assessment

#### Assessment of Motor Symptoms

A dose of 2.5 or 5.0 mg/day of selegiline was started in the 28 drug-naïve PD patients. In patients started on 2.5 mg/day of selegiline, the dose was increased to 5.0 mg/day 1 week later. Within 2 months from the drug initiation, the dose was increased to 7.5 mg/day. However eight patients refused this increase in dose, because they were satisfied with improvements in motor function and activity of daily living. In six patients, the dose was increased to 10 mg/day, the approved maximum dose. Therefore the stable maintenance dose was 5.0–10.0 mg/day. Motor function was assessed using the Unified PD Rating Scale (UPDRS; Movement Disorder Society Task Force on Rating Scales for Parkinson’s Disease, 2003) part III (motor score) before the medication, at the end of each selegiline dose while the doses were increasing, and at 1–2 months after the optimal dose was confirmed (last assessment). Examiners of the UPDRS were blinded to the results of ^123^I-Ioflupane SPECT and the ^123^I-MIBG myocardial scintigraphy, and other clinical information. The score improvement from baseline to the last assessment was assessed as a percent improvement calculated with the following equation:

Percent improvement=score improvement from baseline/baseline UPDRS part III score

#### Assessment of ^123^I-Ioflupane SPECT

^123^I-Ioflupane SPECT was performed using a standard protocol before the dopaminergic medication. A dose of 167 MBq of ^123^I-Ioflupane was injected intravenously into participants. Scanning was performed at 3 h after the injection. All patients were scanned with a triple head gamma camera (GCA-9300R, Toshiba Medical Systems Corporation; Tochigi, Japan) equipped with low energy-high resolution Fan-beam collimators (system resolution at 13.2 cm = 9.6 mm). One hundred and 28 projections for each detector were acquired on a 128 × 128 matrix (1.7 mm pixel size) over a circular 360 orbit. A 20% window centered at 159 KeV was used. The radius of rotation was 13.2 cm for a total acquisition time of 28 min. Image reconstruction was performed by filtered back projection using a Butterworth pre-filter (cut off 0.76 cycles/cm, order 8). Attenuation and scatter corrections were not performed for the image reconstruction. For assessment of the scan, the DatView software (AZE corporation; Tokyo, Japan) was used. The region of interest (ROI) was set at the bilateral putamen and caudate. The entire cerebrum except the above mentioned ROI was used as the reference region with a non-specific tracer binding. The Specific Binding Ratio (SBR) for the striatum was calculated using the following equation:

SBR=[Specific binding(caudate and putamen)−nonspecific binding]/nonspecific binding

The average of the right and left SBR was used in the study.

#### Assessment of ^123^I-MIBG Myocardial Scintigraphy

^123^I-MIBG myocardial scintigraphy was performed as follows. 111 MBq of ^123^I-MIBG was injected intravenously. Myocardial images were acquired using a standard-field gamma camera (Symbia, SIEMENS Healthcare; Tokyo, Japan) equipped with a medial energy collimator. A 15% window centered at 159 KeV was used. Anterior view planar imaging of the chest was performed for 240 s. Identical acquisitions were obtained at 15 min after tracer injection (early images) and at 3 h after tracer injection (delayed images). Images were acquired using 512 × 512 matrices and a 1.0 zoom ratio. To evaluate the myocardial accumulation of ^123^I-MIBG, the H/M ratio was calculated from both the early and delayed images.

### Statistical Analysis

The Kolmogorov-Smirnov test showed the parameters of the SBR, H/M ratio, baseline UPDRS part III score and percent improvement in UPDRS Part III score were normally distributed. Therefore the SBR and H/M ratio were compared with the baseline score, and the percent improvement in UPDRS part III score using Pearson’s correlation coefficients. The level of significance was set at *p* < 0.05 (two-tailed probability).

## Results

**Figure 1:**
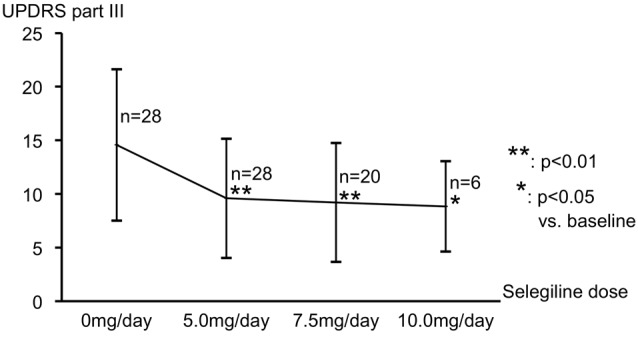
Change from baseline in the UPDRS part III score at each medication dose. Mean UPDRS part III scores significantly improved from baseline with 5.0 mg/day of selegiline. Further improvement in motor function with a dose higher than 5.0 mg was not observed. Abbreviation: UPDRS, Unified Parkinson’s Disease Rating Scale.

Patient backgrounds are shown in Table 1. The dose of selegiline at the last assessment was 5.0 mg/day for eight patients, 7.5 mg/day for 14 patients, and 10.0 mg/day for six patients. No patient decreased his/her dose of selegiline once it was increased to higher dose. The motor symptoms of all patients, except one, improved and satisfaction with activity of daily life (ADL) was achived. Their UPDRS part II (ADL) score significantly decreased from 6.75 ± 3.93 at baseline to 4.36 ± 2.86 at the last assessment (*p* < 0.05). The duration from the drug initiation to the last assessment with the confirmed maintenance dose was from 1 month to 3 months for each patient. Figure 1 shows the change from baseline in the UPDRS part III score at each medication dose. The mean ± standard deviation of this score at baseline and at 5.0, 7.5 and 10.0 mg/day were 14.6 ± 7.0, 9.6 ± 5.4, 9.2 ± 5.5 and 8.8 ± 4.2, respectively. All participants took at least 5.0 mg/day of selegiline, and their mean UPDRS part III score significantly improved. Some patients took more selegiline dose of either 7.5 mg/day (20 patients) or 10.0mg/day (six patients), but further significant improvement over the dose of 5.0 mg/day was not observed.

**Table 1:** Patient backgrounds.

Male:Female	12:16
Age	69.1 ± 9.2 years old
Duration from symptom onset	1.3 ± 0.8 years
Hoehn and Yahr scale	I: 4 cases
	II: 13 cases
	III: 11 cases
Selegiline dose at the last assessment	5.0 mg/day: 8 cases
	7.5 mg/day: 14 cases
	10.0 mg/day: 6 cases

**Figure 2:**
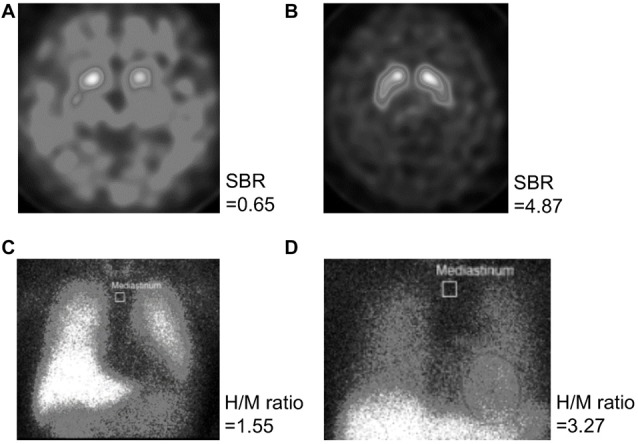
Images of the ^123^I-Ioflupane SPECT and ^123^I-meta-iodobenzylguanidine (MIBG) myocardial scintigraphy. ^123^I-Ioflupane SPECT images of the patients with **(A)** the lowest and **(B)** the highest SBR (average of the right and left sides) and ^123^I-MIBG myocardial scintigraphy images of the patients with **(C)** the lowest and **(D)** the highest H/M ratio of the early image are shown. Abbreviations: SPECT, single photon emission computed tomography; SBR, specific binding ratio for the striatum; H/M ratio, heart-to-mediastinum ratio.

For imaging studies, six patients rejected to undergo both ^123^I-Ioflupane SPECT and ^123^I-MIBG myocardial scintigraphy. Two patients were examined with only ^123^I-MIBG myocardial scintigraphy and the other four patients underwent only ^123^I-Ioflupane SPECT. Figure 2 shows the ^123^I-Ioflupane SPECT images of the patients with (a) the lowest; and (b) the highest SBR (average of right and left sides) and ^123^I-MIBG myocardial scintigraphy images of the patients; with (c) the lowest; and (d) the highest H/M ratio (early image). The SBRs and H/M ratios (early image) of all other participants distributed in these ranges.

**Figure 3:**
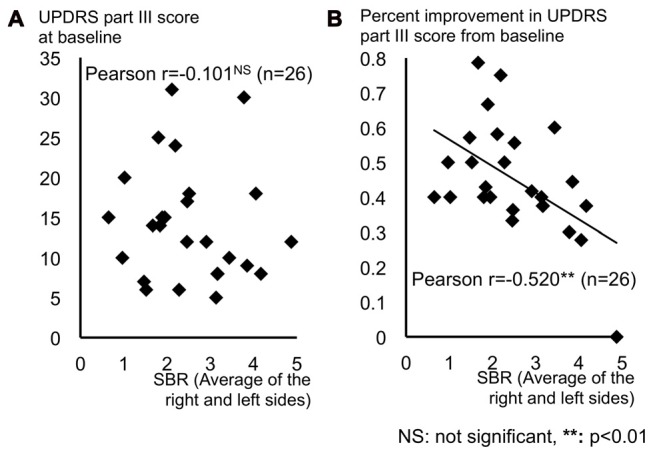
Distribution of SBR (average of the right and left sides) and **(A)** UPDRS part III score at baseline and **(B)** percent improvement in UPDRS part III score from baseline. **(A)** The UPDRS part III score at baseline showed no correlation with the SBR. **(B)** Percent motor improvement from baseline significantly correlated with the SBR. Abbreviations: UPDRS, Unified Parkinson’s Disease Rating Scale; SBR, specific binding ratio for the striatum.

Figure 3 shows distribution of SBR (average of the right and left sides) and (a) UPDRS part III score at baseline; and (b) percent improvement in UPDRS part III score from baseline. The SBR significantly correlated with the percent improvement in UPDRS part III score (Pearson’s *r* = −0.520, *p* < 0.01; Figure 3B), but not with the UPDRS part III score at baseline (Pearson’s *r* = −0.101, *p* = 0.624; Figure 3A). Our patients had different backgrounds, such as age, duration from symptom onset, baseline UPDRS part III score and drug dose, thus we performed a multiple regression analysis to take these factors into account. This analysis showed that percent improvement in UPDRS part III score directly correlated with SBR (*p* = 0.04), but it did not correlate with the age (*p* = 0.72), disease duration (*p* = 0.31), baseline UDRS part III (*p* = 0.77), and the drug dose (*p* = 0.26).

**Figure 4:**
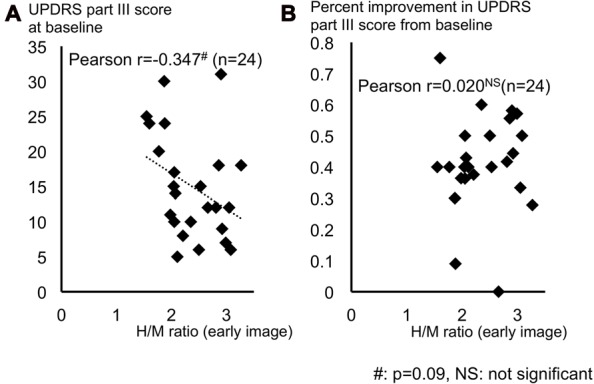
Distribution of H/M ratio of early image and **(A)** UPDRS part III score at baseline and **(B)** percent improvement in UPDRS part III score from baseline. **(A)** The UPDRS part III score at baseline tended to correlate with the H/M ratio (early image). **(B)** Percent improvement in UPDRS part III didn’t correlate with the H/M ratio (early image). Abbreviations: UPDRS, Unified Parkinson’s Disease Rating Scale; H/M ratio, heart-to-mediastinum ratio.

Figure 4 shows distribution of H/M ratio of early image and (a) UPDRS part III score at baseline and (b) percent improvement in UPDRS part III score from baseline. The H/M ratio of early image mildly tended to correlate with the baseline UPDRS part III score (Pearson’s *r* = −0.347, *p* = 0.09), but did not correlate with percent improvement in the UPDRS part III score (Pearson’s *r* = 0.020, *p* = 0.926). The H/M ratio of the delayed image were not significantly correlated with UPDRS part III score at baseline (*r* = −0.159, *p* = 0.457) or with percent improvement (*r* = −0.068, *p* = 0.75).

## Discussion

Neurodegeneration in the striatum of PD patients with a low SBR is estimated to be more severe than in patients with a higher SBR, and their endogenous dopamine is more depleted. This speculation is supported by previous studies using animal models. For example, low 2β-carbomethoxy-3β-(4-[^123^I]iodophenyl)tropane ([^123^I]β-CIT) accumulation in rat brain model correlated with pathological findings, including low dopaminergic fiber density (Bäck et al., 2013) and the pharmacological findings of low dopamine concentration (Saji et al., 2003). In a human study using ^123^I-Ioflupane SPECT, reduced striatal uptake correlated with a lower number of dopaminergic cells (Colloby et al., 2012). Therefore, the effect of selegiline monotherapy was speculated to be weak in the patients with a low striatal ^123^I-Ioflupane uptake because their nigrostriatal dopaminergic nerve degeneration was severe. But in our present study, motor function in patients with a lower SBR showed a greater percent improvement with selegiline monotherapy regardless of baseline UPDRS part III score. One possible explanation for this result is that the improvement was mediated by not only the direct pharmacological effect of selegiline but also by a placebo effect. PD patients sometimes experience a placebo effect. Placebo treatment groups in a clinical trial for PD showed the same treatment efficacy as active medication groups (Diamond et al., 1985). In a clinical study for deep brain stimulation, sham stimulation also showed efficacy (de la Fuente-Fernández, 2004). de la Fuente-Fernández et al. (2001) showed improvement in motor symptoms with simultaneous release of dopamine on the striatum in PD patients taking a placebo, and this indicates that placebo drugs increase nigrostriatal dopamine. Further, they showed that expectation for a reward mediates release of endogenous dopamine from both the ventral striatum and nucleus accumbens while taking placebo drugs (de la Fuente-Fernández et al., 2002). Garris et al. (1999) showed in rat models that expectation for reward mediates dopamine release from the nucleus accumbens, the reward system in the brain. Therefore, expectation for reward, such as symptom relief, mediates the placebo effects by releasing dopamine in the striatum of PD patients (de la Fuente-Fernández et al., 2004).

When patients first begin taking medication, they anticipate symptom relief and may experience a placebo effect. In the placebo group in the DATATOP study examining the efficacy of selegiline (Parkinson Study Group, 1993), patients with symptom improvement had worse baseline motor assessment scores than that of patients with no symptom improvement (Goetz et al., 2002). Shin et al. (2016) showed the placebo effect is greater in patients having more advanced motor symptoms in meta-analysis including placebo groups of 48 clinical studies for PD. Therefore, our present patients with low SBR may have experienced a placebo effect in addition to the real effect of selegiline and had greater symptom improvement.

Another effect of selegiline administration is suspected. Selegiline mildly inhibits dopamine re-uptake at the pre-synaptic dopamine transporter in a rat model (Zsilla et al., 1986) and is shown to be metabolized to L-demethylselegiline, L-methamphetamine, L-amphetamine in the liver (Heinonen et al., 1989). These metabolites increase dopamine release from neurons and inhibit dopamine uptake (Taylor and Snyder, 1970; Lamensdorf et al., 1999; Tekes and Magyar, 2000). Therefore, selegiline administration can increase synaptic dopamine levels. The placebo effects mentioned above may be enhanced by these metabolites.

In addition, there are some compensatory mechanisms against dopaminergic neuron loss in PD patients and animal models. For example, dopamine release from residual striatum neuron is elevated and hyper activity of residual neurons serves to maintain dopaminergic function (Zigmond et al., 1990). In early PD patients, dopamine receptors in post synaptic neurons are up regulated (Hägglund et al., 1987). These compensatory mechanisms have a possibility to have contributed to the effect of selegiline monotherapy with real selegiline’s effect in our patients.

We did not consider a correlation of body weight and the selegiline dose in this observational study for the following reason. The phase I study for selegiline in Japan showed at least 5.0 mg/day of selegiline completely inhibited platelet MAO-B activity in patients with various body weights (Ono et al., 1991). On the other hand, platelet and brain MAO-B activity correlated while taking a MAO-B inhibitor (Bench et al., 1991). Therefore 5.0 mg/day of selegiline completely inhibited brain MAO-B activity in patients included in this phase I study. In our study, significant motor improvement was obtained at 5.0 mg/day, and further dose escalation did not improve motor function. This signifies complete MAO-B inhibition with 5.0 mg/day of selegiline. Furthermore, MAO-B inhibition by selegiline is irreversible. Therefore, continuous daily intake of 5.0 mg/day of selegiline is sufficient to completely inhibit MAO-B activity. The above mentioned results of the Japanese phase I study and our present results demonstrate that this complete inhibition by 5.0 mg/day of selegiline occurs regardless of body weight. In other words, we believe efficacy of selegiline monotherapy is not dose-dependent and can be assessed at the dose of 5.0 mg/day.

We also believe that the selegiline dose is not correlated with the placebo effect. Both placebo and real effects of selegiline may have improved motor symptoms in PD patients, and distinction between these effects is difficult. No previous study has examined the degree of the concomitant placebo effect in treatment using dopaminergic drugs, such as selegiline, to improve Parkinsonian symptoms. The theory that the placebo effect is mediated by expectation for a reward is widely accepted (Lidstone, 2014), but there is no evidence for a correlation of the dose of placebo or drug with the degree of the placebo effect. Expectation-related dopamine release in the nucleus accumbens is an “all or nothing” response (de la Fuente-Fernández et al., 2002). Therefore, our results showing a ceiling effect at 5.0 mg/day support the suggestion that the dose of selegiline did not correlate with the placebo effect.

There are two types of MAO: MAO-A, which metabolizes noradrenaline and serotonin; and MAO-B, which metabolizes dopamine. Selegiline acts as a selective MAO-B inhibitor at low doses, but this selectivity is lost at higher doses: in human studies, selegiline at 10 mg/day showed selective inhibition of MAO-B (Riederer and Youdim, 1986; Fowler et al., 2001), but selegiline at 20 mg or more showed no MAO selectivity (Schulz et al., 1989; Sunderland et al., 1994). Therefore, the dose of selegiline for PD patients in clinical practice is restricted to up to 10 mg/day to prevent side effects caused by effects on non-dopaminergic transmitters. In the present study using 10 mg/day or less of selegiline, the effects of selegiline on PD patients are likely to be dopaminergic, while those mediated by other neurotransmitters were very small.

In our study, the H/M ratio of the early image tended to correlate with baseline motor function. However, Saiki et al. showed that the H/M ratio of the early image correlated with motor severity in PD (Saiki et al., 2004). Other reports show correlation between the H/M ratio and motor severity is controversial (Orimo et al., 2016). Therefore, further studies of this issue are required. Our results show utility of two imaging approaches differs; ^123^I-MIBG myocardial scintigraphy may be a marker of severity of baseline motor function, and ^123^I-Ioflupane SPECT predicts efficacy for selegiline monotherapy in drug-naïve PD patients.

Diagnosis of PD is possible without use of ^123^I-Ioflupane SPECT and ^123^I-MIBG myocardial scintigraphy. However, diagnostic and surrogate biomarkers to confirm clinical diagnosis are required and development of such biomarkers is underway. Currently, these two imaging methods are the only two available biomarkers for pathological and pharmacological findings in PD, and each of these biomarkers may provide information that is not obvious in physical findings. Therefore, it is meaningful to use radiological studies for diagnosis, while considering other application. Although the efficacy of selegiline for motor function may be weaker than that of levodopa and dopamine agonists, selegiline has some clinical benefits. Therefore, responders to this medication should be detected in the early phase. Therefore we believe that ^123^I-Ioflupane SPECT is useful for this purpose.

The SBR differs among institutions and can be compared with effect of selegiline monotherapy within each institute. This study provides a pilot data with regard to usefulness of ^123^I-Ioflupane SPECT. Thus replication studies in larger cohorts and at other institutes are warranted.

## Conclusion

The effect of selegiline monotherapy on improving motor symptoms is greater in drug naïve PD patients with evidence of more severe impairment in the nigrostriatal dopaminergic neurons using ^123^I-Ioflupane SPECT. Efficacy of selegiline monotherapy may be mediated by not only selegiline’s direct activity but also other beneficial effects, such as a placebo effect and compensate mechanism against striatal dopaminergic loss.

## Author Contributions

The study design and concept was done by HM and YOwan. Acquisition, analysis and interpretation of data were done by all the authors. Drafting of the manuscript was done by HM. All the authors revised the manuscript critically for important intellectual content. Statistical analysis was done by HM and YOwan. All the authors contributed significantly to the latter version of the manuscript and approved the final version of the manuscript.

## Conflict of Interest Statement

The authors declare that the research was conducted in the absence of any commercial or financial relationships that could be construed as a potential conflict of interest.
